# *Pthlh*, a promising cancer modifier gene in rat tongue carcinogenesis

**DOI:** 10.3892/or.2013.2859

**Published:** 2013-11-20

**Authors:** HIROHIKO SUWA, MASATO HIRANO, KOUJI KAWARADA, MOTOHIKO NAGAYAMA, MICHIKO EHARA, TOMONARI MURAKI, HAYASE SHISA, AIKO SUGIYAMA, MASAHIRO SUGIMOTO, HIROSHI HIAI, MOTOO KITANO, JUN-ICHI TANUMA

**Affiliations:** 1 Department of Oral Pathology, Division of Oral Pathogenesis and Disease Control, Asahi University School of Dentistry, Mizuho, Gifu, Japan; 2 Department of Pathology, Saitama Cancer Center Research Institute, Ina, Saitama, Japan; 3 Malignancy Control Research Laboratory, Medical Innovation Center, Graduate School of Medicine, Kyoto University, Sakyo-Ku, Kyoto, Japan; 4 Division of Pathology, Saitama Cooperative Hospital, Kawaguchi, Saitama, Japan

**Keywords:** *Pthlh*, PTHrP, tongue cancer, speed congenic rat, microarray analysis

## Abstract

Susceptibly to the induction of rat tongue cancer (TC) by oral 4-nitroquinoline 1-oxide (4NQO) exposure is a polygenic trait. Among several quantitative trait loci identified by crosses between TC-susceptible Dark Agouti (DA) rats and TC-resistant Wistar-Furth (WF) rats, we focused on tongue cancer susceptibility locus (*Tcas3*) of chromosome 4. We examined tongue carcinogenesis in the reciprocal congenic strains DA.WF-*Tcas3* and WF.DA-*Tcas3* and in their parental strains. The *Tcas3^DA^* allele, and not the *Tcas3^WF^* allele, significantly favored tumor latency, incidence and TC number/size. In genomic DNA of TCs induced in (DA × WF) F1 rats, the resistant *Tcas3^WF^* allele was frequently and selectively lost, particularly in larger tumors. Thus, we searched the possible candidate genes in the *Tcas3* region using microarray analysis of TCs in F1 rats and revealed significant upregulation of 2 cancer-related genes, parathyroid hormone-like hormone (*Pthlh*) and *Kras2*. The relevance of the WF allele of *Pthlh* as a cancer modifier was indicated by 3 single nucleotide polymorphisms specific to this strain. In contrast, no consistent strain-specific variations were found in *Kras2*. Moreover, the plasma Ca^2+^ level was consistently higher in DA rats when compared to the level in WF rats bearing TCs; moreover, the *Pthlh*-mRNA expression level was >30-fold higher in TCs when compared to this level in the normal tongue mucosa. Immunostaining experiments showed strong PTHrP protein expression in TCs of DA rats, and the signal was intensified in larger TCs. *Kras2* was also upregulated in TCs, but to a lesser degree than PTHrP. Thus, *Pthlh* is a promising candidate modifier gene in the development and progression of rat TCs.

## Introduction

In our previous research, we showed that susceptibility to 4-nitroquinoline 1-oxide (4NQO)-induced rat tongue carcinogenesis is a polygenic trait involving a number of susceptibility and resistance quantitative trait loci (QTLs) (1,2). Dark Agouti (DA) rats are highly susceptible to 4NQO-induced tongue cancer (TC), whereas Wistar-Furth rats (WF) are very resistant (3,4). A genome-wide association study in F2 progeny of these 2 strains revealed 5 significant QTLs, namely *Tscc1-5* (*Tcas1-5* in the Rat Genome Database), which account for differences in susceptibility to TC (2,5,6).

In the present study, we focused on the single-locus effects of the tongue cancer susceptibility QTL 3 (*Tcas3*) on rat chromosome 4 (RNO4) by constructing reciprocal speed congenic strains; WF rats carrying a DA-derived *Tcas3* (*Tcas3^DA^*) chromosomal segment and DA rats carrying a WF-derived *Tcas3* (*Tcas3^WF^*) chromosomal segment. The modifier effects of *Tcas3* on 4NQO-induced tongue carcinogenesis were confirmed by allele type-dependent incidence of TCs and frequent deletion of *Tcas3^WF^* in TCs in (DA × WF) F1 rats. Subsequently, we identified genes responsible for these modifying effects from the *Tcas3* region by comparing microarray analyses of TCs and normal tongue tissues, revealing a significant elevation in parathyroid hormone-like hormone (*Pthlh)* and *Kras2* expression. Immunohistochemistry of TCs showed that the *Pthlh* signal was more intense than the *Kras2* signal. Subsequent sequencing of DNA showed 3 unique single-nucleotide polymorphisms (SNP) in the WF *Pthlh* gene. Rats carrying the *Tcas3^DA^* and 4NQO-induced TCs had elevated serum *Pthlh* and Ca^2+^ levels and their WF *Pthlh* gene carried 3 single-nucleotide polymorphisms not found in other rat strains. These findings suggest that *Pthlh* is a promising candidate gene, located at *Tcas3, involved* in the development and progression of rat TC.

## Materials and methods

### Animals

The following strains of rats and their F1 (DA × WF) progeny were used in the present study. Dark Agouti/SIc rats were purchased from Japan SLC Inc. (Hamamatsu, Japan); Fisher 344/DuCrj (F344) rats were from Charles River Japan Inc. (Atsugi, Japan); Sprague-Dawley/JcI (SD) rats were from Japan Clea Co. (Tokyo, Japan); and DRH rats were from SEAC Yoshitomi (Fukuoka, Japan). Long Evans/Stm (LE) rats were introduced from the Saitama Cancer Center (Ina, Japan); ACI/Ms (ACI) rats were from the National Institute of Genetics (Mishima, Japan); and Donryu rats were from Osaka University (Osaka, Japan). The Wistar-Furth/Stm (WF) rats were originally from Hiroshima University (Hiroshima, Japan). The present study was carried out according to the Animal Care Guidelines of Asahi University, Gifu, Japan (10-007).

### Generation of speed congenic lines

Speed congenic strains for the *Tcas3* locus were developed using marker-assisted selection starting from an intercross between DA and WF rats (7,8). The chromosomal segment containing *Tcas3* was defined by microsatellite markers *D4Rat20*6 and *D4Rat72*. A male rat of F1 with DA-derived *Tcas3* (*Tcas3^DA^*) from an F1 × WF cross was selected and mated with WF females (8,9). After serial selective backcrossing with WF rats over 6 generations, heterozygous littermates were mated. Then a pair of littermates that were homozygous for *Tcas3^DA^* were selected and subjected to successive inbreeding. The resulting strain was designated WF.DA-*Tcas3*. The reciprocal congenic strain DA.WF-*Tcas3* was generated using the same mating protocol. All congenic rats used in the present study were of the third or subsequent generation of *Tcas3* homozygotes.

### Genotype analysis for speed congenic lines

All primers for PCR-based microsatellite analysis were purchased from Research Genetics, Inc. (Huntsville, AL, USA). We used 135 of the 360 polymorphic markers between DA and WF rats to characterize congenic strains. Of these, 40 were found on RNO4 and the others were distributed 10–30 cM apart on each rat chromosome (5,6). Theoretically, the percentage of DA/WF segments was <1%. PCR and agarose electrophoresis of PCR products were performed as described previously (1,2,10).

### Tongue cancer induction

A stock solution of 4NQO (Nacalai Tesque Inc., Kyoto, Japan) at a concentration of 200 mg/l in 5% ethanol was prepared and stored at 4°C until use. Starting at 6 weeks of age, all rats were given drinking water containing 0.001% 4NQO *ad libitum* from 5 p.m. to 9 a.m. Rats were inspected once a day for TC development and were weighed once a week. All of the rats given 4NQO (DA, WF, F1 and congenic rats) were sacrificed when they became moribund or on day 180 of the experiment. Full necropsy and histopathological examinations were performed. The diameter of the largest TC tumor (DTCmax) and the number of TCs with a diameter ≥5 mm (TC#5) were recorded at necropsy. Paired samples of the largest TC and kidney or tail from each rat were obtained and stored at −80°C. High-molecular-weight DNA was extracted from the frozen tissues as previously described (1,2).

### Loss of heterozygosity (LOH) analysis

LOH in the *Tcas3* region, including that of *Kras2* (RGD ID: 5036392) and *Pthlh* (RGD ID: 11187) genes of induced TCs in F1 rats was determined using PCR-based microsatellite analysis (1,2). Fluorescently tagged primers were purchased from Applied Biosystems (Foster City, CA, USA). Positions of loci were mapped on the Rat Genome Database (6). LOH analysis was performed for tumor samples from the F1 progeny of DA and WF rats as previously described (11,12). Genomic DNA from TCs of 100 reciprocal F1 rats (50 females and 50 males) was used to survey LOH. Samples were scanned using a PRISM 310 Genetic Analyzer, and the data were collected automatically and analyzed using GeneScan software (both from Applied Biosystems). A Genotyper 2.0 was used to score alleles and assess LOH. A difference between the alleles was expressed as the ratio of the tumor signal to the normal signal (T1/T2 over N1/N2). Ratios <0.67 or >1.35 were considered indicative of LOH for that locus (12).

### Microarray analysis

TCs >5 mm in diameter and normal tissues from F1 rats were analyzed using microarrays. RNA was isolated from the tissues and subjected to linear amplification by RiboAmp RNA Amplification kit (Arcturus, Mountain View, CA, USA). RNA amplification efficiency was compared with that of control RNA of known quantity (0.1 μg) by running in parallel with the 18 samples. Gene expression analysis was performed using the GeneChip^®^ Rat Gene 1.0 ST Array (Affymetrix Inc., Santa Clara, CA, USA) technique according to the manufacturer’s protocol. The arrays were scanned, and fluorescence intensity was measured using Microarray Suite v5.0 software (Affymetrix Inc.). The arrays were then imported into DNA-Chip analyzer software for normalization and model-based analysis. All statistical analyses were performed using S-Plus 6.0 (Insightful, Seattle, WA, USA) software. Three criteria were applied to determine differentially expressed gene transcripts as follows. First probe sets on the array that were assigned as ‘absent’ call in all samples. Second, a two-tailed Student’s t-test was used for comparison of the average gene expression signal intensity between the F1 TCs (TC, 5–10 mm; n=15) and normal samples (n=3), and differences were considered significant at a critical α level of 0.05. Finally, fold ratios were calculated for gene transcripts that showed a statistically significant difference (P<0.05), and only gene transcripts that exhibited >2-fold changes were included in further analyses.

### Single-strand conformation polymorphism (SSCP) analysis and sequencing in F1 TC rats

To detect mutations in genes of the *Tcas3* region including *Pthlh* and *Kras2*, SSCP analysis was performed in tumor samples from F1 progeny of DA and WF rats, as previously described (12,13). Samples with altered electrophoretic mobility were re-amplified, and direct sequencing of both strands was performed to confirm and characterize mutations.

### Quantitative real-time PCR

Total RNA was extracted from the tumors and normal tongue mucosa from 4NQO-induced TCs from 50 F1 rats. Tissue specimens were homogenized using an RNAqueous kit (Ambion, Grand Island, NY, USA). cDNA was generated using a High Capacity cDNA Transcription kit (Ambion) and was amplified by PCR using a TaqMan Universal PCR Master Mix and TaqMan Gene Expression assays with 18S (Rn03928990-g1), *Pthlh* (Rn00561818-g1) and *Kras2* (Rn00580460-m1) (Applied Biosystems). PCR products were analyzed using Applied Biosystems StepOne™ with Gene Amp software and StepOne real-time PCR systems (13).

### Direct sequence analysis of Pthlh in 8 rat strains

To detect SNPs in rat *Pthlh* (NC-005103.3, ID24695, M34108), genomic DNA was obtained from the rat kidney, and the entire sequence of the *Pthlh* locus was determined in 8 rat strains using an ABI 310 Genetic Analyzer (Applied Biosystems, Foster City, CA, USA). Allele calling was performed using the Genotyper 2.0 software (Applied Biosystems), and identified SNPs were compared with those previously reported (12–15).

To determine whether SNPs in translational regions led to structural or functional amino acid substitutions in the *Pthlh* protein, we conducted *in silico* analysis using freely available software, including MutPred (16), Panther (17), PhD-SNP (18), SNAP (19), PolyPhen2 (20) and SIFT (21). In addition, we evaluated the impact of SNPs on transcription factor-binding sites by searching for potential regulatory motifs at the 5′ UTR using Matlnspector (22) and TESS (23). The software default values were used in all bioinformatic analyses.

### Plasma electrolytes, serum Pthlh-related proteins and cytokines

Plasma electrolytes including Ca^2+^, Na^+^, K^+^, IP and Cl^−^ were determined at Japan SLC, Inc. (24). The serum levels of Pthlh-N (normal <3.9 pmol/l), Pthlh-intact (normal <1.1 pmol/l) and Pthlh-C (normal <55.3 pmol/l), and interleukin (IL)-6, -8 and -11 (SRL Inc., Tokyo, Japan) were also determined (25).

### Immunohistochemistry of tongue cancer

All tongue specimens from F1 rats were routinely fixed in buffered 10% formalin and were embedded in paraffin. Serial 4-μm sections were dewaxed in xylene and rehydrated in graded ethanol. Sections were stained with hematoxylin and eosin (H&E) to confirm histological diagnosis, and separate sections were used for immunohistochemical analyses. Sections were incubated with diluted rat polyclonal anti-PTHrP antibody (sc-9685; 1:100) and mouse monoclonal anti-Kras2A antibody (sc-13794; 1:50) (both from Santa Cruz Biotechnology, Inc., USA) as primary antibodies overnight at 4°C.

### Statistical analysis

Numerical data are presented as means ± standard deviation (SD). Statistical analyses were performed using one-way ANOVA or Fisher’s test with SPSS 17.0 (SPSS Inc., Chicago, IL, USA) software.

## Results

### Generation of speed congenic rat strains for Tcas3

**Figure 1:**
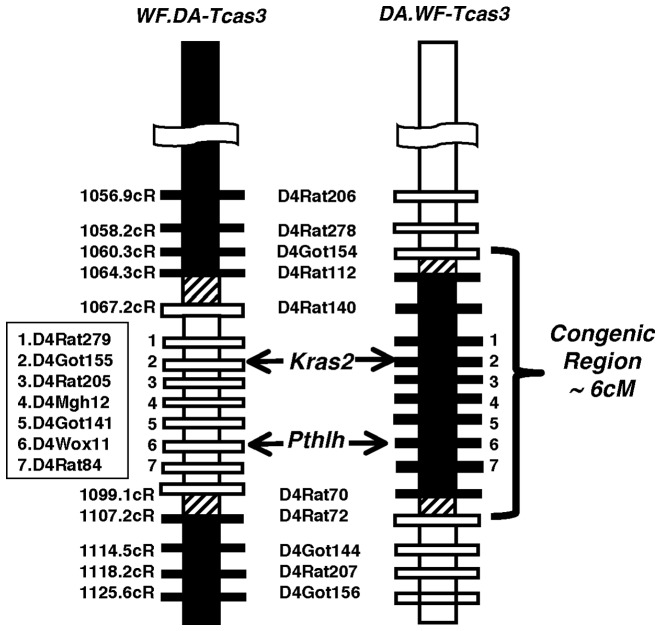
*Tcas3* segment of RNO4 in the WF.DA-*Tcas3* and DA.WF-*Tcas3* rats. The closed column represents a WF-derived segment, the hatched column is undetermined and the open column represents a DA-derived segment. The closed bars represent the loci with the WF allele, and open bars represent loci with the DA allele. Numbers on the left indicate the map location of marker loci listed in the box (CR). RNO4, rat chromosome 4; WF, Wistar-Furth rats; DA, Dark Agouti rats.

To study the phenotypic effects of the single *Tcas3* locus on TC development, we established a set of speed congenic strains for *Tcas3* using marker-assisted backcrossing of DA and WF rats and generated the WF.DA-*Tcas3* and DA.WF-*Tcas3* strains, respectively (8,9). WF.DA-*Tcas3* rats had an introgressed DA-derived RNO4 segment spanning D4Rat140 to D4Rat70, whereas DA.WF-*Tcas3* rats carried a WF-derived RNO4 segment spanning D4Rat112 to D4Rat70 (Fig. 1). Both segments were ~6-cM long, were partly overlapping and contained several cancer-related genes including *Pthlh* and *Kras2* (Fig. 1).

### Effects of Tcas3 on tongue carcinogenesis

**Figure 2:**
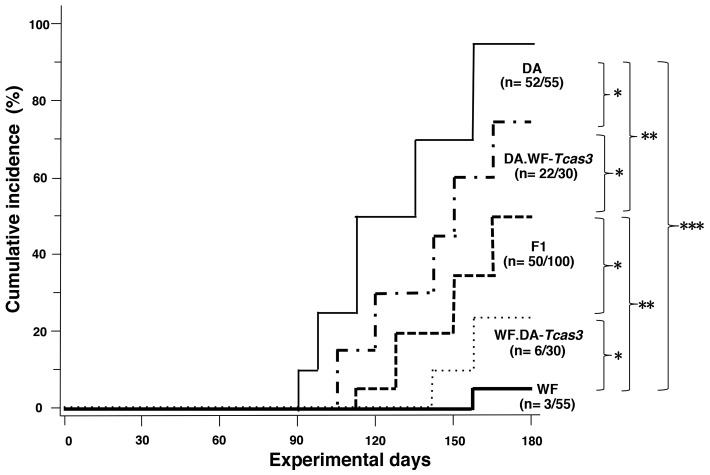
Cumulative incidence of 4NQO-induced tongue cancers (TC#5, >5 mm). The solid line represents WF, the dotted line WF.DA-*Tcas3*, the dashed lines F1, the dotted/dashed lines DA.WF-*Tcas3* and the thin solid line DA rats. ^*^P<5×10^−2^ significantly different from the corresponding values for WF and WF.DA-*Tcas3*, DA and DA.WF-*Tcas3*; ^**^P<5×10^−2^ for DA and WF; F1 and WF; ^***^P<1×10^−4^ for WF and DA. 4NQO, 4-nitroquinoline 1-oxide; TC, tongue cancer; WF, Wistar-Furth rats; DA, Dark Agouti rats.

We previously reported tumor incidence, number and size in descending order in DA, F1 and WF rats (1,2,12). To evaluate the effect of introgressed *Tcas3* segments, rats of both congenic strains, parental DA and WF strains and F1, were administered 4NQO, and tongue and oral carcinogenesis was observed. As shown in Fig. 2, DA rats developed TCs with the shortest latency and highest incidence, whereas WF rats developed TCs with the longest latency and lowest incidence. In congenic strains, *Tcas3^DA^* showed modest but significant accelerating effects on TCs in WF rats, whereas *Tcas3^WF^* had TC-inhibitory effects on DA rats. These differences in strains were reflected by incidence, number and size of tumors (Table I). These observations clearly indicate that *Tcas3* alone exhibits significant phenotypic effects on the development and growth of TCs and oral cancers. That is *Tcas3^DA^* augmented grown whereas *Tcas3^WF^* suppressed it.

### Selective LOH of Tcas3^WF^ in larger TCs

**Figure 3:**
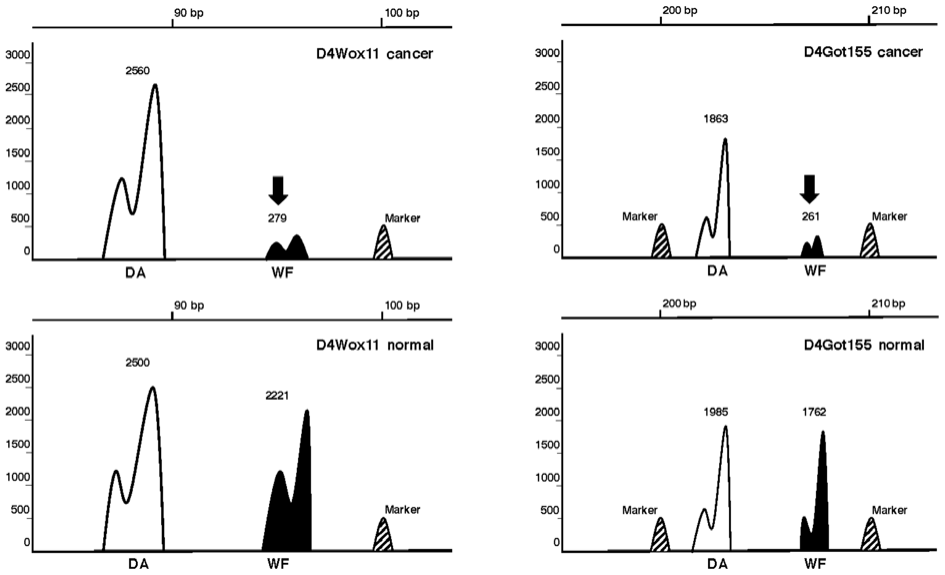
Representative LOH data analysis at microsatellite loci D4Wox11 (*Pthlh*) (left) and D4Got155 (right). The upper panels show the peak heights in arbitrary units of the WF (filled) and DA (unfilled) alleles from a TC in a (DA × WF) F1 rat, and the lower panels show those from the normal tongue tissue of the same rat. LOH, loss of heterozygosity; WF, Wistar-Furth rat; DA, Dark Agouti rat.

To determine whether suppressive *Tcas3^WF^* was selectively deleted in TCs, genomic DNA of TCs induced in F1 rats was examined for LOH. Out of 100 F1 rats, 10 TCs of <5-mm in diameter, 29 TCs of 5–10 mm in diameter and 40 non-TC tissues did not show any LOH. However, in 21 TCs of ≥10-mm diameter, LOH was observed between D4Mgh10 and D4Mgh13 on chromosome 4q44. In the majority of cases, the WF allele was selectively lost (Fig. 3). Among the major cancer-related genes on the *Tcas3* segment, the incidence of LOH at *Kras2* and *Pthlh* was 32.7 and 40.8%, respectively in the TC#5 (Table II). Loss of the *Tcas3^WF^* allele only in larger TCs indicates that it represents a late event in tumor progression.

### Microarray analysis

To identify genes that are responsible for the effects of *Tcas3*, microarray analysis was performed in TCs and normal tongue tissues from F1 rats. According to the Rat Genome Database (6), 27 genes, 6 ESTs and 18 pseudogenes are present in the *Tcas3* region. Table III shows the expression of the 27 genes as determined by microarray analysis. Significantly increased expression was observed for *Pthlh* (7.33-fold, P<0.001) and slightly less for *Kras2* (5.21-fold, P<0.001).

**Figure 4:**
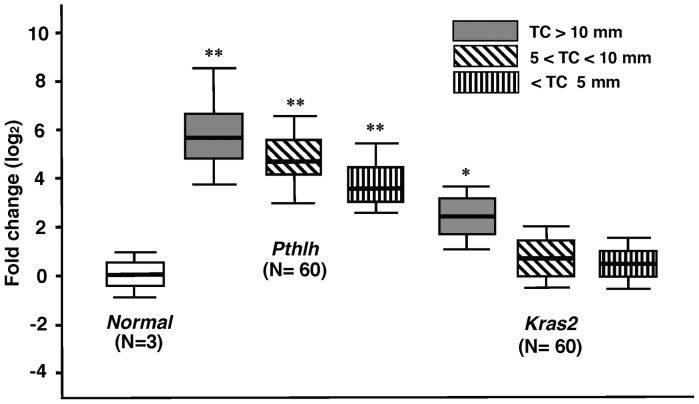
Quantitative analysis of *Pthlh* and *Kras2* mRNA levels in F1 tongue cancer (TC): TC >10 mm, TC 5–10 mm, TC <5 mm and non-TC tissue F1 samples. The line within each box represents the median fold change. The upper and lower edges of each box represent the 75th and 25th percentiles, respectively. The upper and lower bars represent the highest and lowest value determined, respectively. ^**^P<0.001, ^*^P<0.01.

The expression of genes in the *Tcas3* region was analyzed in 10 control and 60 TC samples using quantitative real-time RT-PCR. *Pthlh* mRNA expression in large TCs (>10 mm) was consistently >30-fold higher than that in normal tongue mucosa (Fig. 4), and expression levels were higher in larger tumors.

### Sequencing of cancer-related genes in the Tcas3 segment

**Figure 5:**
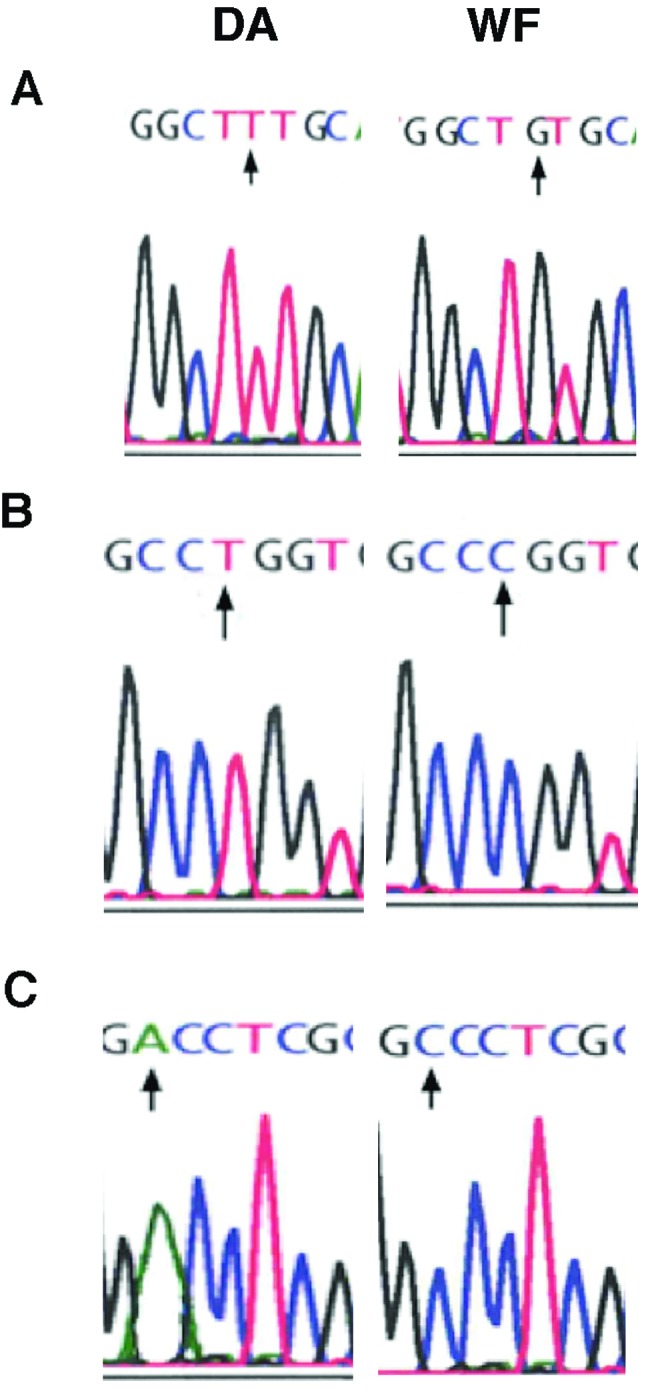
Detection of sequence variants in the *Pthlh* gene in the WF and DA strains. The WF strain contained 3 SNPs not noted in the DA and 6 other inbred strains of rat. The SNPs were located at positions (A) +2 bp, (B) +17 bp and (C) +513 bp. The sequences of the primers used for PCR amplification before sequencing were: *Pthlh* 1 F, 5′-GACTCGCTCACT TCTCAGCA-3′ and R, 5′-GGCTCCCATAGCAATGTCTA-3′; *Pthlh* 2 F, 5′-GCGGTGTCTGAGCACCAGCTA-3′ and R, 5′-GCACAGCGGACAGAC AATACC-3′. Primer pair 1 was used to amplify the SNPs at positions +2 and +17 bp; pair 2 amplified the SNP at position +1485 bp (B). WF, Wistar-Furth rats; DA, Dark Agouti rats.

*Pthlh* and *Kras2* were further examined by sequencing germline and/or tumor DNA. Direct sequencing of the germline *Pthlh* gene was performed for 8 laboratory rat strains: DA, LE, SD, ACI, Fischer 344, Donryu, DRH and WF. The *Pthlh* gene in resistant WF rats was found to carry 3 SNPs at positions +2 bp (T→G), +17 bp (T→C) and +1485 (A→C) from the 5′ end of exon 1, but these were not observed in the other strains (Fig. 5). The base change at position 2 and 17 does not cause amino acid substitutions, whereas that at position +1485 [496 bp in the coding sequence (CDS)] bp would substitute the polar residue threonine with the hydrophobic residue proline at the 166th amino acid (ACC→CCC) of the precursor protein (position 130th of the mature protein) in which the CDS start is at 527 bp.

The potential impact of the 3 SNPs was determined using computational analyses. Since 2 SNPs at +2 bp (T→G) and +17 bp (T→C) were found in the untranslated region of the *Pthlh* gene, we searched for known transcription factor-binding sites within the −950 to 526 bp region. TCF-4E (CTTTGCA) and ZFX (GAGGCCTGGTG) motifs were found at −1 to +6 bp and +11 to +21 bp, respectively, in sequences from DA, LE, SD, ACI, F344, Donryu and DRH rats in which the +2 bp and 17 bp positions were occupied by T. In the sequence from WF rats, PLU1-JARID1B.01 (TGGCTGTGC), SP1 (TGTGC) and ROAZ.01 (GTGCACCCAGAGGCCCG) motifs were found at −4 to +5 bp, +1 to +5 bp and +2 to +8 bp, respectively, with G and C residing at +2 bp and +17 bp, respectively. The agreement rates in matrices of sequence motifs were 100, 98.8, 96.6, 100 and 73.1%, respectively.

Using computational analyses, we evaluated the impact of the 166th amino acid substitution on the structure and function of the Pthlh protein. MedPred predicted a low impact, with a probability of deleterious mutation score of 0.134 (>0.5 indicates impact). Panther also predicted that the substitution was almost neutral, with a deleterious probability of 0.34315 (>0.5 indicates impact) and PhD-SNP predicted no impact. SNAP also predicted that the impact was neutral with 85% expected accuracy. Albeit with low confidence, SHIFT gave a score of 0 for affected protein function. Polyphen2 did not yield a prediction. Thus, all bioinformatics tools revealed almost no impact of these SNPs on Pthlh protein structure and function.

Subsequently, *Kras2* point mutations were examined at codons 12, 13 and 61 in F1 TCs. One of the tumors had a heterozygous CAA→CAT mutation in codon 61 of *Kras2*, resulting in an amino acid substitution from glutamine to histidine. No other activating mutations were found in the *Kras2* gene. Therefore, any involvement of *Kras2* may not be predominant in rat TCs.

### Plasma electrolyte and antibody levels in the WF, congenic and DA rats

Table IV shows the titer of plasma electrolytes and immunoreactive PTHrP in control and TC-bearing rats. Electrolyte levels in control rats did not vary according to genetic background and *Tcas3* genotype. However, plasma Ca^2+^ levels were consistently higher in TC-bearing rats than in control rats. We also evaluated levels of PTHrP peptides derived from post-translational cleavage of the whole protein. The PTHrP-C peptide levels were significantly elevated in TC-bearing rats, whereas levels of PTHrP-N and PTHrP-intact were unchanged.

### TC immunohistochemistry

**Figure 6:**
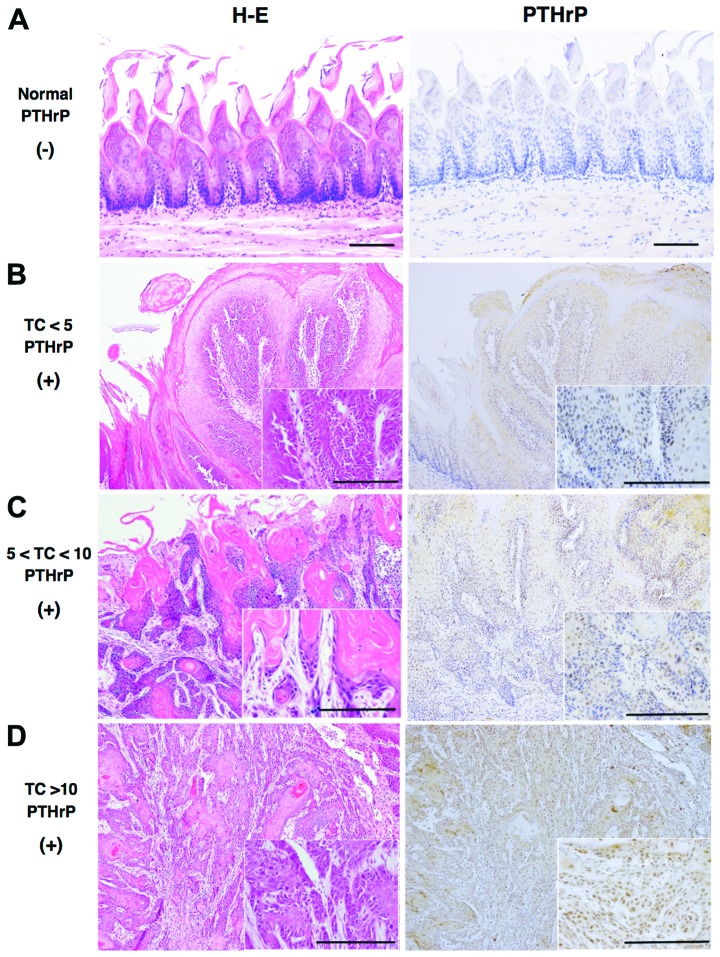
Hematoxylin and eosin (H&E) staining (left) and anti-PTHrP immunohistochemistry (right) of F1 normal rat tongue (A) and tongue cancers (TC) (<5 mm in diameter (TC <5) for H&E and PTHrP-positive (+) (B); TC of 5–10 mm (TC 5–10) for H&E and PTHrP (+) (C); TC >10 mm (TC >10) for H&E and PTHrP (+) (D). The PTHrP-(+) tissue shows a positive signal in the cytoplasm and nucleus of many cancer cells. Scale bars, 200 μm.

Among 50 TC#5 and 10 rats with TCs of <5 mm in F1 rats, positive signals for PTHrP were detected in the nucleus and cytoplasm of prickle-type cancer cells (Fig. 6). Staining intensity above or below the cut-off score (10%) was classified as ‘positive’ or ‘negative’, respectively, at a magnification of ×100 using computer-associated image analyzer software (WinROOF 7.1; Mitani Co., Japan) (26). As shown in Table V, the fraction of positively stained TCs increased with their size. There were significant differences in TCs between the TC#5 and DTCmax groups (P<0.001), and among the TCs <5 mm, 5–10 mm and TC >10 mm (P<1×10^−10^, χ^2^=47.63). The expression of *Kras2* was consistently weaker than that of PTHrP (data not shown).

## Discussion

Out of 5 QTLs that affect susceptibility to TCs in 4NQO-treated rats, we focused on *Tcas3* in the present study. In genome-wide association studies in the F2 rat, the peak logarithm of the odds score 6.88 was observed for *Tcas3* at 4q44 (2). This chromosomal region is homologous to human 12p12.1-q11.2 and harbors the cancer-related genes *Pthlh* and *Kras2*(7,12). To evaluate the effects of a single *Tcas3*, we generated speed congenic strains for *Tcas3* using marker-assisted backcrossing.

As shown in Table I and Fig. 2, the quantitative parameters of carcinogenesis, namely tumor latency, incidence, tumor number and size, were moderately but significantly modified by *Tcas3*. Hence the *Tcas3^DA^* allele increased susceptibility to tongue carcinogenesis and progression, whereas the *Tcas3^WF^* allele bestowed resistance. Selective loss of the resistance allele *Tcas3^WF^* was frequent in larger TCs induced in F1 rats, and the consensus stretch of LOH contained *Pthlh* and *Kras2*. Microarray comparisons of TCs and normal tongue mucosa also indicated that among genes in the *Tcas3* region these 2 genes were significantly highly expressed. We focused on *Pthlh* following the observation that resistant WF *Pthlh* rats carry 3 unique SNPs. The impact of these SNPs will be discussed in greater detail below. Other observations that suggest the relevance of *Pthlh* in tongue carcinogenesis include (i) a 30-fold higher expression of *Pthlh* in TCs than that in normal tongue epithelium, (ii) very strong PTHrP immonostaining in larger TCs from F1 rats and (iii) increased plasma levels of Ca^2+^ and PTHrP-C in TC-bearing DA rats when compared with the control rats.

The anabolic effects of *Pthlh* have been demonstrated in rodents and in humans. *Pthlh* is expressed in many cell types and usually acts as an autocrine, paracrine, and/or intracrine factor that plays numerous roles in embryonic development and physiology (27,28). PTHrP may also have important functions in tumor development. PTHrP has been shown to stimulate proliferation and invasiveness of cancer cells as well as protection from apoptosis. PTHrP has the potential to cause malignant hypercalcemia and induce local osteolysis, which facilitates the growth of tumor cells that have metastasized to bone (28,29). Partial homology of PTHrP and parathyroid hormone allows PTHrP to activate parathyroid hormone 1 receptor (PTH1R). PTH1R activation in bone and kidney leads to bone absorption and renal calcium retention, respectively, inducing a rise in blood calcium levels (30).

PTHrP consists of 3 molecular domains, the parathyroid hormone-like domain in the N-terminal region, a mid-region and a C-terminal domain. Post-translational cleavage of the PTHrP protein allows these domains to function independently. The parathyroid hormone-like domain stimulates protein kinase A, protein kinase C, and/or the calcium-dependent pathways by activating PTH1R (31,32). The mid-region domain can enter the nucleus since it carries a bipartite nuclear localization sequence at residues 88–91 and 102–106 and an importin β-binding site at residues 66–94 (33). After translocation into the nucleus, the PTHrP mid-region domain modulates gene expression by an as yet undefined mechanism. Nuclear transport of the mid-region domain is regulated by CDK1(CDC2)/CDK2-dependent phosphorylation (34). The C-terminal domain of PTHrP (osteostatin) physically interacts with β-arrestin (35), which regulates internalization and desensitization of ligand-stimulated G-protein-coupled receptors (36). Osteostatin bears a number of phosphorylation sites that are important for the mitogenic activity of PTHrP in vascular smooth muscle cells (37).

To understand the potential impact of SNPs in untranslated regions of PTHrP genes, we performed *in silico* analyses using computational tools with various prediction algorithms and found several known regulatory motifs on the 5′-UTR of the *Pthlh* sequence. These analyses identified only 2 transcription factors, TCF-4E and ZFX, in all strains apart from WF, indicating that the SNPs at +2 bp (T→G) and +17 bp (T→C) eliminate the transcriptional factor binding site. In contrast, PLU1-JARID1B.01, SP1 and ROAZ.01 were found only in WF rats, indicating that these 2 SNPs affect transcription factor binding to these motifs. ZFX and ROAZ.01 are zinc finger proteins with a DNA-binding motif, and SP1 is common to many eukaryotic transcriptional regulatory pathways. The transcription factor TCF-4E regulates expression of members of the Wnt pathway and its splicing isoforms are related to the progression of renal cell carcinoma (38). The motif PLU1-JARID1B.01 was previously identified in the development and progression of breast cancer (39). However, no clear relationship between these transcription factors and oral cancer development has been reported to date. Hence, further research is necessary to understand the effect of these motifs and SNPs at +2 and +17 bp. Using several bioinformatics tools with varying predictive algorithms, we also analyzed structural and functional effects of the SNP at +1485 bp, which leads to an amino acid substitution at the 166th amino acid on the Pthlh protein. Notably, none of these analyses suggested that this SNP would have any effect. Potentially, these computational tools, which are generated using characterized protein, may not predict novel functional motifs, even though they yielded consistent predictions. Benelli *et al*(15) reported an amino acid polymorphism in *Pthlh* that elicited cancer-modifying effects in a mouse squamous cell carcinoma cell line (15). Importantly, the SNP at position +1485 [+496 bp in the coding sequence (CDS)] bp was common to both mouse and rat models. This SNP can be expected to substitute the polar residue threonine with the hydrophobic residue proline at the 166th amino acid (ACC→CCC) of the precursor protein (position 130th of the mature protein), in which the CDS start is at 527 bp.

The present study revealed that *Tcas3* alone contributes moderate but significant phenotypic effects to 4NQO-induced rat tongue carcinogenesis. Several observations suggest that *Pthlh* is a promising candidate gene in Tcas3, although further studies using appropriate reporter assays and *in vivo* transgenic analyses are required to provide direct supporting evidence of this. In subsequent studies, we will focus on structural and functional analyses of *Pthlh* and its products.

## Figures and Tables

**Table I:** 4NQO-induced rat tongue and other cancers in the WF, F1, congenic and DA rats.

	WF (n=55)	Congenic WF.DA-*Tcas3* (n=30)	F1 (n=100)	Congenic DA.WF-*Tcas3* (n=30)	DA (n=55)
Rats with TC#5 (incidence, %)	5.5 (3/55)	20 (6/30)^b^	50 (50/100)^c^	73.3 (22/30)^c^	94.6 (52/55)^d^
No. of TC#5^a^	0.23±0.22	0.33±0.12^b^	0.61±0.71^c^	0.68±1.20^c^	1.28±1.56^d^
Total no. of TCs^a^	0.52±0.82	0.75±0.58^b^	0.92±0.86^c^	1.01±0.76^c^	1.59±0.66^d^
DTCmax (mm)	1.18±1.63	2.12±3.54^b^	5.15±1.85^c^	5.89±1.17^c^	12.2±4.49^d^
Total no. of cancers^a^	1.26±1.45	1.39±5.46^b^	1.56±1.67^c^	2.89±1.64^c^	3.45±1.58^d^

4NQO, 4-nitroquinoline 1-oxide; TC, tongue cancer; WF, Wistar-Furth rats; DA, Dark Agouti rats. TC#5, number of TCs ≥5 mm in diameter. DTCmax, diameter of the largest TC. Total no. of cancers include TC and other cancers (the hard palate, pharynx, larynx, gingiva, trachea and esophagus).

a Number/rat;

b significantly different from the corresponding value for WF rats (5×10^−2^>p>1×10^−3^);

c significantly different from the corresponding value for WF rats (1×10^−3^>p>1×10^−4^);

d significantly different from the corresponding value for WF rats (p<1×10^−4^).

**Table II:** Immunostaining of the PTHrP protein in tongue cancer (TC) samples from F1 rats.

Rat case no.	DTCmax (mm)	TC#5	Microsatellite markers^a^ on RNO4										

			*Mgh30*	*Mgh10*	*ENO2*	*Mit27*	*Rat140*	*Kras2*	*Got155*	*Pthlh*	*Rat70*	*Rat72*	*Mgh13*
30	10	2	-	-	-	-	-	-	-	WF	-	-	-
31	12	1	-	-	-	-	-	-	-	WF	-	-	-
32	12	2	-	-	-	-	-	-	WF	WF	-	-	-
33	13	1	-	-	-	-	-	-	WF	WF	-	-	-
34	13	1	-	-	-	-	-	WF	WF	WF	-	-	-
35	14	1	-	-	-	-	-	WF	WF	WF	-	-	-
36	14	2	-	-	-	-	-	WF	WF	WF	-	-	-
37	15	2	-	-	-	-	-	WF	NI	WF	-	-	-
38	15	2	-	-	-	-	-	WF	WF	WF	WF	-	-
39	15	2	-	-	-	-	-	WF	WF	WF	WF	-	-
40	16	1	-	-	-	-	-	NI	WF	WF	WF	-	-
41	17	2	-	-	-	-	-	WF	WF	WF	WF	-	-
42	18	1	-	-	-	-	-	WF	WF	WF	WF	-	-
43	19	2	-	-	-	-	-	DA	DA	DA	WF	WF	-
44	20	3	-	-	-	-	WF	WF	WF	WF	WF	WF	-
45	21	2	-	-	-	-	WF	WF	WF	WF	WF	WF	-
46	22	2	-	-	-	-	WF	WF	WF	NI	WF	WF	-
47	23	2	-	-	-	-	WF	WF	WF	WF	WF	WF	-
48	24	2	-	-	-	-	WF	WF	WF	WF	WF	WF	-
49	25	2	-	-	-	WF	WF	WF	WF	WF	WF	WF	WF
50	25	3	-	WF	WF	WF	WF	WF^b^	WF	WF	WF	WF	WF
Incidence			0/50	1/50	1/50	2/50	7/50	16/49	18/49	20/49	13/50	8/50	2/50
Total (%)			0	2.0	2.0	4.0	14.0	32.7	36.7	40.8	26.0	16.0	4.0

RNO4, rat chromosome 4; 4NQO, 4-nitroquinoline 1-oxide; TC, tongue cancer. The lost alleles are denoted: WF, Wistar-Furth; DA, Dark Agouti; -, the presence of 2 alleles; NI, not informative. DTCmax, maximum diameter of the largest TC; TC#5; number of TCs ≥5 mm in diameter.

a Loci are shown in their relative order on RNO4 from the centromere (left) to the centromere (right);

b *Kras2* mutation.

**Table III:** Microarray analysis of the *Tcas3* region between tongue cancer and normal tissues in F1 rats.

Symbol	Accession no.	Gene name	Fold change	P-value
*Abcc9*	NM-013040.2	*ATP-binding cassette, subfamily C (CFTR/MRP), member 9*	−1.58	0.413
*Aebp2*	NM-001106626.1	*AE binding protein 2*	0.23	0.625
*Bcat1*	NM-017253.2	*Branched chain amino acid transaminase 1, cytosolic*	−2.11	0.073
*Bicd1*	NM-001108653.1	*Bicaudal D homolog 1*	2.31	0.071
*Capza3*	NM-017164.1	*Capping protein (actin filament) muscle Z-line, α3*	−2.72	0.061
*Casc1*	BG377837.1	*Cancer susceptibility candidate 1*	2.29	0.085
*Cmas*	NM-001009419.1	*Cytidine monophosphate N-acetylneuraminic acid synthetase*	1.71	0.561
*Fgfr1op2*	NM-201421	*FGFR1 oncogene partner 2*	2.89	0.051
*Gys2*	NM-013089.1	*Glycogen synthase 2*	1.12	0.761
*Iapp*	NM-012586	*Islet amyloid polypeptide*	0.56	0.982
*Itpr2*	NM-031046.3	*Inositol 1,4,5-trisphosphate receptor, type 2*	1.26	0.781
*Kcnj8*	NM-017099.4	*Potassium inwardly-rectifying channel, subfamily J, member 8*	1.54	0.564
*Kras2*	NM-031515.1	*Kirsten rat sarcoma viral oncogene homologue 2*	5.21	<0.001
*Ldhb*	NM-012595.1	*Lactate dehydrogenase B*	1.89	0.647
*Mgst1*	NM-134349.2	*Microsomal glutathione S-transferase 1*	−2.54	0.062
*Pde3a*	NM-017337.1	*Phosphodiesterase 3A, cGMP inhibited*	1.98	0.653
*Pik3c2g*	NM-053923.1	*Phosphatidylinositol-4-phosphate 3-kinase, catalytic subunit type 2γ*	2.01	0.076
*Plekha5*	AI009219.1	*Pleckstrin homology domain-containing protein family A, member 5*	1.51	0.711
*Pthlh*	NM-012636.1	*Parathyroid hormone-like peptide*	7.33	<0.001
*Ptpro*	NM-017336.1	*Protein tyrosine phosphatase, receptor type, O*	1.09	0.881
*Rassf8*	NM-001191753	*Ras association domain family (N-terminal) member 8*	2.97	0.051
*Slc21a6*	NM-130736.1	*Solute carrier organic anion transporter family, member 1a6*	0.12	0.979
*Slco1a2*	NM-030838.1	*Solute carrier organic anion transporter family, member 1A2*	0.18	0.957
*Slco1c1*	NM-053441.1	*Solute carrier organic anion transporter family, member 1c1*	0.16	0.969
*Sox5*	NM-001014060	*SRY (gender determining region Y)-box 5*	1.11	0.652
*St8sia1*	NM-012813.2	*ST8 α-N-acetyl-neuraminide α-2,8-sialyltransferase 1*	1.87	0.489
*Tm7sf3*	NM-001011970.1	*Transmembrane 7 superfamily member 3*	−2.32	0.065

**Table IV:** Plasma electrolyte and antibody levels in the WF, congenic and DA rats.

Rat	Cancer	No. of rats	Na^+^ (mEq/l)	K^+^ (mEq/l)	Ca^2+^ (mEq/l)	IP (mg/dl)	Cl^−^ (mEq/l)	PTHrP N-terminal (pmol/l)	PTHrP intact (pmol/l)	PTHrP C-terminal
WF	No	52	139.9±0.72	2.89±0.41	5.58±0.11	2.68±1.56	101.1±0.57	<3.9	<1.1	55.4±0.45
	TC	3	140.1±0.22	2.92±0.26	6.71±0.16	2.89±0.66	102.3±0.98	<3.9	<1.1	68.4±1.76^a^
WF.DA-*Tcas3*	No	24	140.6±0.53	2.91±0.35	5.59±0.27	2.91±0.67	101.3±0.39	<3.9	<1.1	57.4±0.55
	TC	6	142.1±0.12	3.07±0.86	6.81±0.32^a^	3.59±0.48	102.3±0.11	<3.9	<1.1	70.4±3.76^b^
DA.WF-*Tcas3*	No	8	139.1±0.13	2.97±0.17	5.51±0.17	3.39±0.67	101.2±0.09	<3.9	<1.1	58.4±1.77
	TC	22	142.2±0.63	3.05±0.85	6.91±0.17^a^	4.27±4.49^a^	102.8±0.19	<3.9	<1.1	131.4±0.47^b^
DA	No	3	136.1±0.15	2.56±0.57	5.59±0.24	2.15±1.38	100.1±0.10	>3.9	>1.1	74.4±0.48
	TC	52	137.8±0.46	3.06±0.68	7.89±0.65^a^	5.45±1.59^b^	103.4±0.61^a^	>3.9	>1.1	149.4±0.88^b^

Serum levels of electrolyte data referenced by Japan SLC, Inc.

a Significant compared to the value for control rats (1×10^−3^>p>1×10^−4^);

b significant compared to the value for control rats (p<1×10^−4^) by ANOVA analysis.

WF, Wistar-Furth; DA, Dark Agouti; TC, tongue cancer.

**Table V:** Immunostaining of the PTHrP protein in tongue cancer (TC) samples from F1 rats.

	Positive	Negative
Rat normal tissue (n=10)	0	10
TCs <5 mm (n=10)	1	9
TCs 5–10 mm (n=29)	24	5
TCs >10 mm (n=21)	21	0

Staining intensity above or below the cut-off score (10%) was classified as ‘positive’ or ‘negative’ at ×100 magnification using a computer-associated image analyzer software (WinROOF 7.1). Significantly different from the corresponding values for F1 normal rats tissue (P<1×10^−10^, χ^2^=47.63).
